# Autoregulation of blood flow drives early hypotension in a rat model of systemic inflammation induced by bacterial lipopolysaccharide

**DOI:** 10.1093/pnasnexus/pgad014

**Published:** 2023-01-21

**Authors:** Eduardo H Moretti, Abner C Rodrigues, Bruno V Marques, Leonardo T Totola, Caroline B Ferreira, Camila F Brito, Caroline M Matos, Filipe A da Silva, Robson A S Santos, Luciana B Lopes, Thiago S Moreira, Eliana H Akamine, Luiz A Baccala, André Fujita, Alexandre A Steiner

**Affiliations:** Departamento de Imunologia, Instituto de Ciencias Biomedicas, Universidade de Sao Paulo, Av. Prof. Lineu Prestes, 1730, Sao Paulo, SP 05508-000, Brazil; Instituto Internacional de Neurociencias Edmond e Lily Safra, Instituto de Ensino e Pesquisa Alberto Santos Dumont, Macaiba, RN 59288-899, Brazil; Departamento de Farmacologia, Instituto de Ciencias Biomedicas, Universidade de Sao Paulo, Sao Paulo, SP 05508-000, Brazil; Departamento de Fisiologia e Biofisica, Instituto de Ciencias Biomedicas, Universidade de Sao Paulo, Sao Paulo, SP 05508-000, Brazil; Departamento de Farmacologia, Instituto de Ciencias Biomedicas, Universidade de Sao Paulo, Sao Paulo, SP 05508-000, Brazil; Department of Neurobiology, University of Pittsburgh School of Medicine, Pittsburgh, PA 15213-2548, USA; Departamento de Imunologia, Instituto de Ciencias Biomedicas, Universidade de Sao Paulo, Av. Prof. Lineu Prestes, 1730, Sao Paulo, SP 05508-000, Brazil; Departamento de Imunologia, Instituto de Ciencias Biomedicas, Universidade de Sao Paulo, Av. Prof. Lineu Prestes, 1730, Sao Paulo, SP 05508-000, Brazil; Departamento de Fisiologia e Biofisica, Instituto de Ciencias Biologias, Universidade Federal de Minas Gerais, Belo Horizonte, MG 31270-901, Brazil; Departamento de Fisiologia e Biofisica, Instituto de Ciencias Biologias, Universidade Federal de Minas Gerais, Belo Horizonte, MG 31270-901, Brazil; Departamento de Farmacologia, Instituto de Ciencias Biomedicas, Universidade de Sao Paulo, Sao Paulo, SP 05508-000, Brazil; Departamento de Fisiologia e Biofisica, Instituto de Ciencias Biomedicas, Universidade de Sao Paulo, Sao Paulo, SP 05508-000, Brazil; Departamento de Farmacologia, Instituto de Ciencias Biomedicas, Universidade de Sao Paulo, Sao Paulo, SP 05508-000, Brazil; Departamento de Engenharia de Telecomunicacoes e Controle, Escola Politecnica, Universidade de Sao Paulo, Sao Paulo, SP 05508-900, Brazil; Departamento de Estatistica, Instituto de Matematica e Estatistica, Universidade de Sao Paulo, Sao Paulo, SP 05508-090, Brazil; Departamento de Imunologia, Instituto de Ciencias Biomedicas, Universidade de Sao Paulo, Av. Prof. Lineu Prestes, 1730, Sao Paulo, SP 05508-000, Brazil

**Keywords:** endotoxic shock, septic shock, critical care, blood pressure, vascular function, vasoplegia, perfusion

## Abstract

Uncontrolled vasodilation is known to account for hypotension in the advanced stages of sepsis and other systemic inflammatory conditions, but the mechanisms of hypotension in earlier stages of such conditions are not clear. By monitoring hemodynamics with the highest temporal resolution in unanesthetized rats, in combination with ex-vivo assessment of vascular function, we found that early development of hypotension following injection of bacterial lipopolysaccharide is brought about by a fall in vascular resistance when arterioles are still fully responsive to vasoactive agents. This approach further uncovered that the early development of hypotension stabilized blood flow. We thus hypothesized that prioritization of the local mechanisms of blood flow regulation (tissue autoregulation) over the brain-driven mechanisms of pressure regulation (baroreflex) underscored the early development of hypotension in this model. Consistent with this hypothesis, an assessment of squared coherence and partial-directed coherence revealed that, at the onset of hypotension, the flow–pressure relationship was strengthened at frequencies (<0.2 Hz) known to be associated with autoregulation. The autoregulatory escape to phenylephrine-induced vasoconstriction, another proxy of autoregulation, was also strengthened in this phase. The competitive demand that drives prioritization of flow over pressure regulation could be edema-associated hypovolemia, as this became detectable at the onset of hypotension. Accordingly, blood transfusion aimed at preventing hypovolemia brought the autoregulation proxies back to normal and prevented the fall in vascular resistance. This novel hypothesis opens a new avenue of investigation into the mechanisms that can drive hypotension in systemic inflammation.

**Significance Statement**

Hypotension is a serious complication of sepsis and other systemic inflammatory conditions. It is generally attributed to a malfunction of blood vessels. However, given the dynamic nature of such conditions in experimental models, other mechanisms might be involved. The present study shows that early development of hypotension in a rat model of systemic inflammation does not result from vascular malfunction, but rather from a physiological competition between the local mechanisms of blood flow regulation (tissue autoregulation) and the brain-driven mechanisms of pressure regulation (baroreflex). In this case, edema-associated hypovolemia leads to a heightened gain of tissue autoregulation, which acts to maintain blood flow at the expense of a reduced blood pressure.

## Introduction

Septic shock is a significant cause of mortality worldwide (1, 2). It is currently defined as a condition in which sepsis is complicated by circulatory, cellular, and metabolic abnormalities, having as a hallmark the occurrence of hypotension (3). Progress at reducing mortality in sepsis and other systemic inflammatory conditions has been slow. Nevertheless, it is becoming gradually evident that outcome is being positively impacted by refinement in supportive care (4, 5). We propose that further refinement in supportive care might be achievable by exploring the fact that sepsis-associated hypotension is not a uniform phenomenon, as evidenced by the fact that only half of the patients with this condition are responsive to infusion of crystalloid fluids (6, 7). Distinct mechanisms are probably at play in these clinically distinct patient subsets. To date, however, mechanistic studies on septic hypotension have focused primarily on the advanced, fluid-refractory phase characterized by uncontrolled vasodilation (vasoplegia), heterogeneous organ perfusion, and an increased risk of myocardial depression (8–10). More information is warranted regarding the mechanisms of the fluid-responsive, presumably earlier, phase of hypotension in sepsis.

In experimental animals, challenge with bacterial lipopolysaccharide (LPS) induces systemic inflammation associated with relatively rapid changes in cardiovascular parameters. This model may therefore be suitable to probe new mechanistic aspects of early septic shock. However, studies pertaining to the mechanisms of early LPS-induced hypotension have yielded controversial observations. For example, whereas there are reports of early hypotension (<6 h from the LPS challenge) being associated with decreased blood flow and increased vascular resistance in rats (11, 12) and pigs (13, 14), there are also reports of it being associated with the opposite hemodynamic profile in the same species (15, 16). This raises the question as to whether early hypotension consists of hemodynamically distinct phases. In the previous studies, transition from one hemodynamic state to another might have been overlooked due to infrequent measurements, made at intervals of 30–60 min.

In the present study, we employed the latest technologies in pressure and flow monitoring to track with the highest temporal resolution arterial blood pressure (AP), systemic blood flow (SBF), and systemic vascular resistance (SVR) in freely moving, unanesthetized rats challenged with a hypotension-inducing dose of LPS. This experimental preparation uncovered rapid transitions and three hemodynamically distinct phases in the first 120 min of the response to LPS. The second of these phases corresponded to the development of hypotension. It was characterized by a fall in SVR and a recovery from a previously reduced SBF. We then investigated the mechanisms of this phase by assessing vascular fitness, sympathetic output, blood viscosity, and autoregulation of blood flow. Frequency-domain analyses of spontaneous AP-SBF rhythms aided in the assessment of autoregulation, as did the vasoconstrictor escape phenomenon.

## Results

### Dynamic hemodynamic transitions take place in the early stage of LPS-induced systemic inflammation

**Fig. 1.**
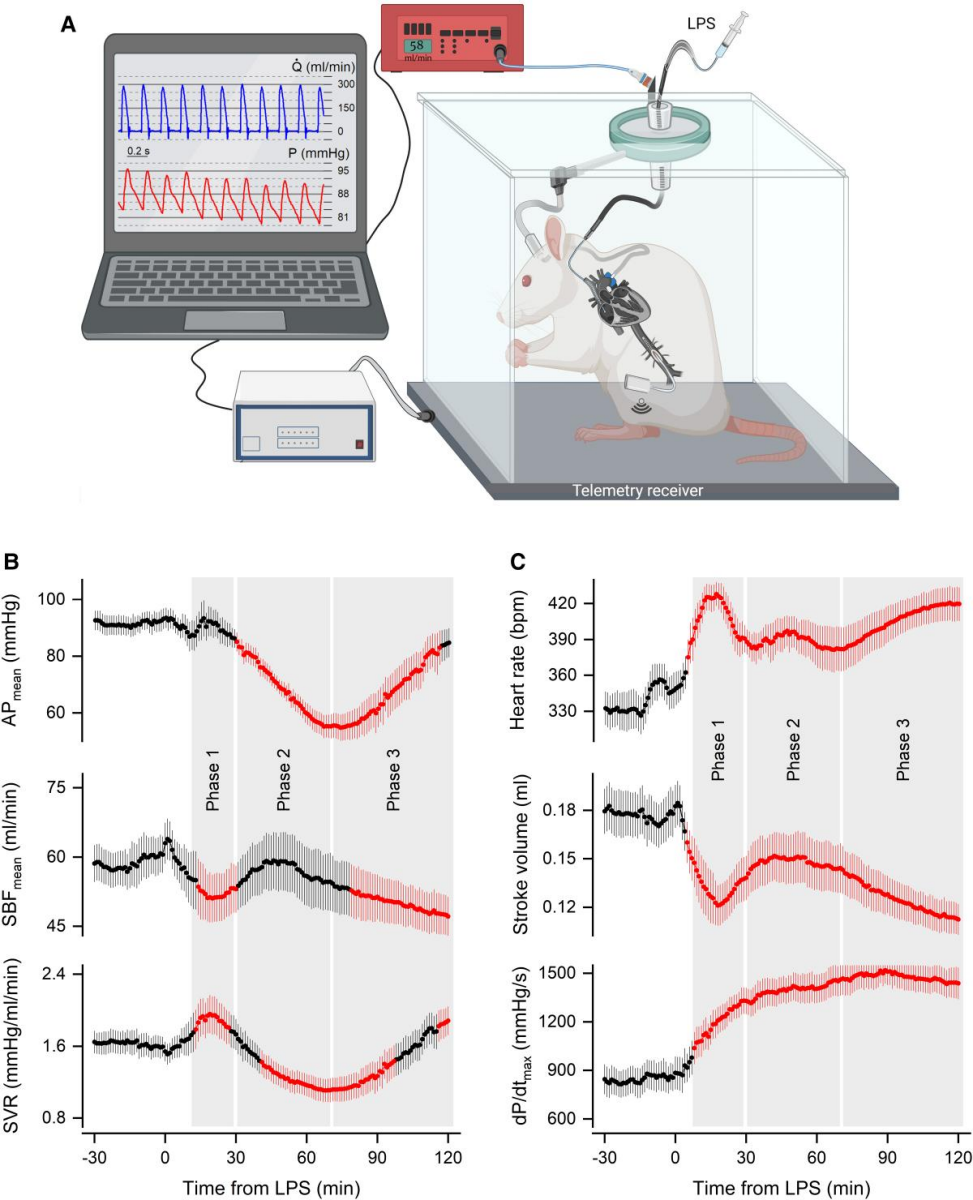
Transitions in systemic hemodynamics during the first 2 h of a challenge with LPS at a relatively high dose (1 mg/kg, i.v.). (A) Schematic representation of the experimental setup used to record hemodynamic parameters with the highest temporal resolution from unanesthetized, freely moving rats. For a description, see Methods and Results. The image was created with BioRender.com. (B) Time courses of the primary hemodynamic parameters: mean arterial pressure (AP_mean_), mean systemic blood flow (SBF_mean_), and systemic vascular resistance (SVR). (C) Time courses of the SBF determinants: heart rate, stroke volume, and d*P*/d*t*_max_ (an index of myocardial contractility). Data are expressed as mean ± SEM; *n* = 11 rats. The red symbols and lines denote statistical differences in relation to the pre-LPS period.

As schematically represented in Fig. 1A, rats prepared for the experiment had a transit-time flow probe implanted around the ascending aorta, a telemetric pressure sensor implanted nonocclusively in the abdominal aorta, and a venous catheter advanced to the right atrium via the jugular vein. A telemetric receiver positioned under the cage captured the signal emitted by the pressure sensor. Both the cable extension of the flow probe and the extension of the venous catheter were passed by swivel systems, thus allowing hemodynamic parameters to be monitored and LPS to be injected while the rat was freely moving and undisturbed by the experimenter.

This experimental preparation revealed three well-defined hemodynamic phases only in the 120 min that followed a challenge with LPS (1 mg/kg, i.v.). The first phase was observed from 10 to 30 min post-LPS and consisted of a drop in SBF and an increase in SVR (Fig. 1B). The drop in SBF was brought about by a decrease in stroke volume, while heart rate and d*P*/d*t*_max_ (an index of myocardial contractility) increased (Fig. 1C). On average, AP maintained at the pre-LPS level, as the drop in SBF was compensated by the increase in SVR. However, in 36% of the animals, a fall in AP was observed prior to the compensatory rise in SVR (Supplementary Material Appendix, Fig. S1).

The second hemodynamic phase was manifest from 30 to 70 min post-LPS. It was characterized by a downward trend in SVR, which went from elevated to decreased in relation to baseline (Fig. 1B). Such a decrease in SVR was associated with a 40% reduction in AP, reaching an average value of 55 mmHg at the nadir of the response (Fig. 1B). Another important characteristic of this phase was that the decreases in SVR and AP were associated with a return of SBF to normality (Fig. 1B). The full recovery in SBF resulted from a partial recovery in stroke volume combined with a second wave of tachycardia (Fig. 1C).

The third hemodynamic phase was observed from 70 to 120 min post-LPS. It corresponded to the recovery from hypotension (Fig. 1B). But although AP and SVR returned to baseline level during this phase, this was not a return to normality, because it resulted in another drop in SBF (Fig. 1B). SBF dropped as the result of a decrease in stroke volume, which was more pronounced than in the first phase (Fig. 1C). Rises in heart rate and d*P*/d*t*_max_ were not sufficient to prevent SBF from falling (Fig. 1C).

### Vasoplegia is not associated with the decrease in SVR during the onset of hypotension

Because hypotension developed in the second hemodynamic phase, we sought to investigate the mechanisms of this phase in more detail. Our first step was to verify whether vasoplegia could underlie the downward trend in SVR. We reasoned that if vasoplegia were a factor in this phase, we should be able to observe an impairment in the intrinsic contractile properties of arterioles as early as 30 min post-LPS. This point in time was when AP began to significantly differ from baseline (Fig. 1B), and it also corresponded to the maximal rate of decrease in SVR (Supplementary Material Appendix, Fig. S2).

**Fig. 2.**
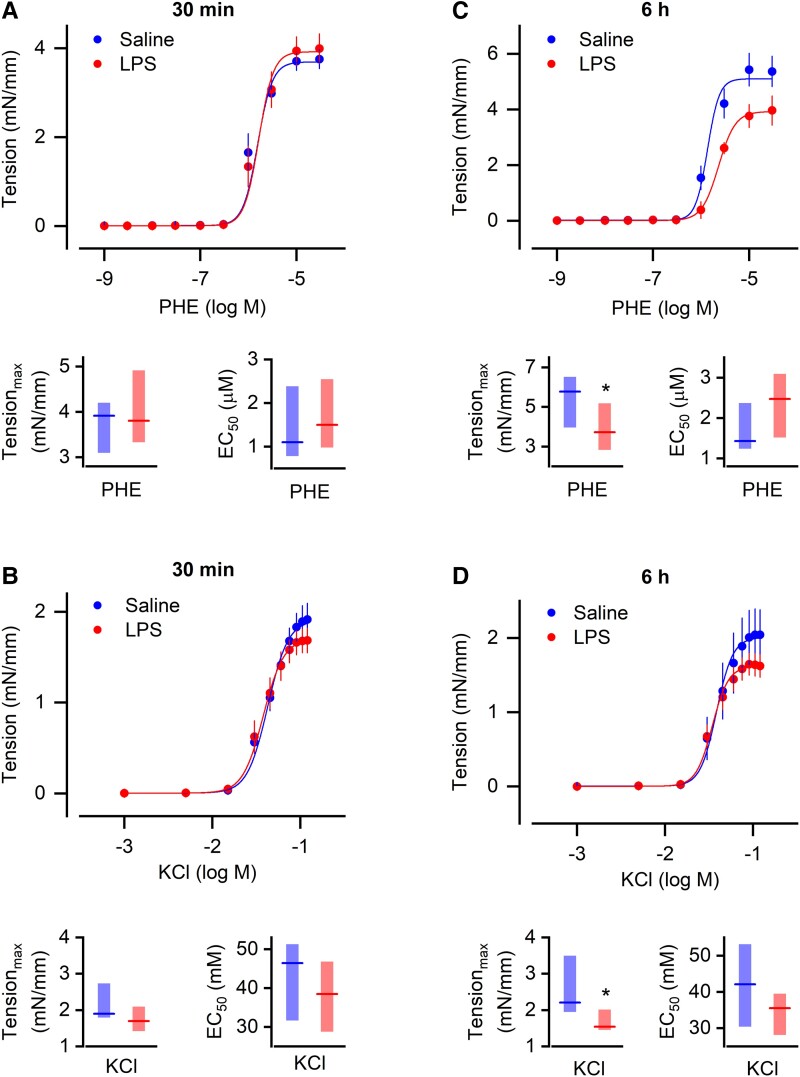
The intrinsic contractile properties of resistance mesenteric arteries are preserved at the time corresponding to the onset of hypotension (30 min post-LPS), even though they are impaired at a later stage (6 h post-LPS). (A and B) Concentration-dependent responses to phenylephrine (PHE, A) or potassium chloride (KCl, B) in mesenteric arteries harvested 30 min after the injection of LPS or saline. (C and D) Concentration-dependent responses to PHE (C) or KCl (D) in mesenteric arteries harvested 6 h after the injection of LPS or saline. In each panel are displayed the concentration-dependent tension curves, as well as the corresponding response maxima (Tension_max_) and EC_50_. Concentration-dependent curves are plotted as mean ± SEM. Tension_max_ and EC_50_ data often failed to meet a normal distribution and are expressed as median (horizontal line) and 95% confidence interval (floating bar). Five to eight arteries from different rats were tested with each vasoactive agent. *Statistical difference between the LPS- and the saline-injected groups.

The mesentery was harvested 30 min after injection of LPS or its vehicle (saline), and third-order branches of the superior mesenteric artery (resistance branches) were evaluated by isometric-tension myography. In this preparation, the α_1_-adrenergic agonist, phenylephrine (PHE), caused isometric contraction in a typical dose-dependent fashion. Nevertheless, such an effect did not differ between arteries harvested from LPS- or saline-injected rats (Fig. 2A). The contractile response to KCl-induced membrane depolarization was similarly unaffected by the LPS challenge (Fig. 2B). Neither the magnitude of the responses nor EC_50_ differed between the groups (Fig. 2A and B). We also evaluated the contractile response to serotonin and the dilatory response to acetylcholine, none of which were impaired at 30 min post-LPS (Supplementary Material Appendix, Fig. S3). Hence, it seems unlikely that vasoplegia could account for the falls in SVR and AP that begin as early as 30 min after a challenge with LPS in rats.

In view of these observations, it became important to confirm that our wire myograph setup would be capable of detecting vasoplegia if it were present. Such a confirmation was conducted in mesenteric arteries harvested at a later stage of LPS-induced systemic inflammation, more specifically, at 6 h after the i.v. injection of LPS. At this later stage, mesenteric arteries from the LPS-challenged rats displayed impaired contractile responses to PHE (Fig. 2C) and, to a lesser extent, to KCl (Fig. 2D). Such an impairment was primarily due to a reduction in the maximal vasoconstriction attained (Fig. 2C and D), which is in agreement with previous studies (17–19).

### The early fall in SVR occurs in the setting of increased sympathetic output

In acute hemorrhage, it is known that SVR can fall early and in the absence of vasoplegia as a result of a brain-driven suppression in sympathetic output (20). Therefore, we hypothesized that a similar phenomenon could be involved in LPS-induced hypotension. To test this hypothesis, we recorded the electrical activity of nerves whose sympathetic fibers play essential roles in cardiovascular regulation: the splanchnic nerve; the lumbar nerve; and the renal nerve. Because it is extremely difficult to obtain chronic nerve recordings in freely moving animals, we chose to perform nerve recordings under anesthesia. All other experiments of the study were conducted in unanesthetized rats.

**Fig. 3.**
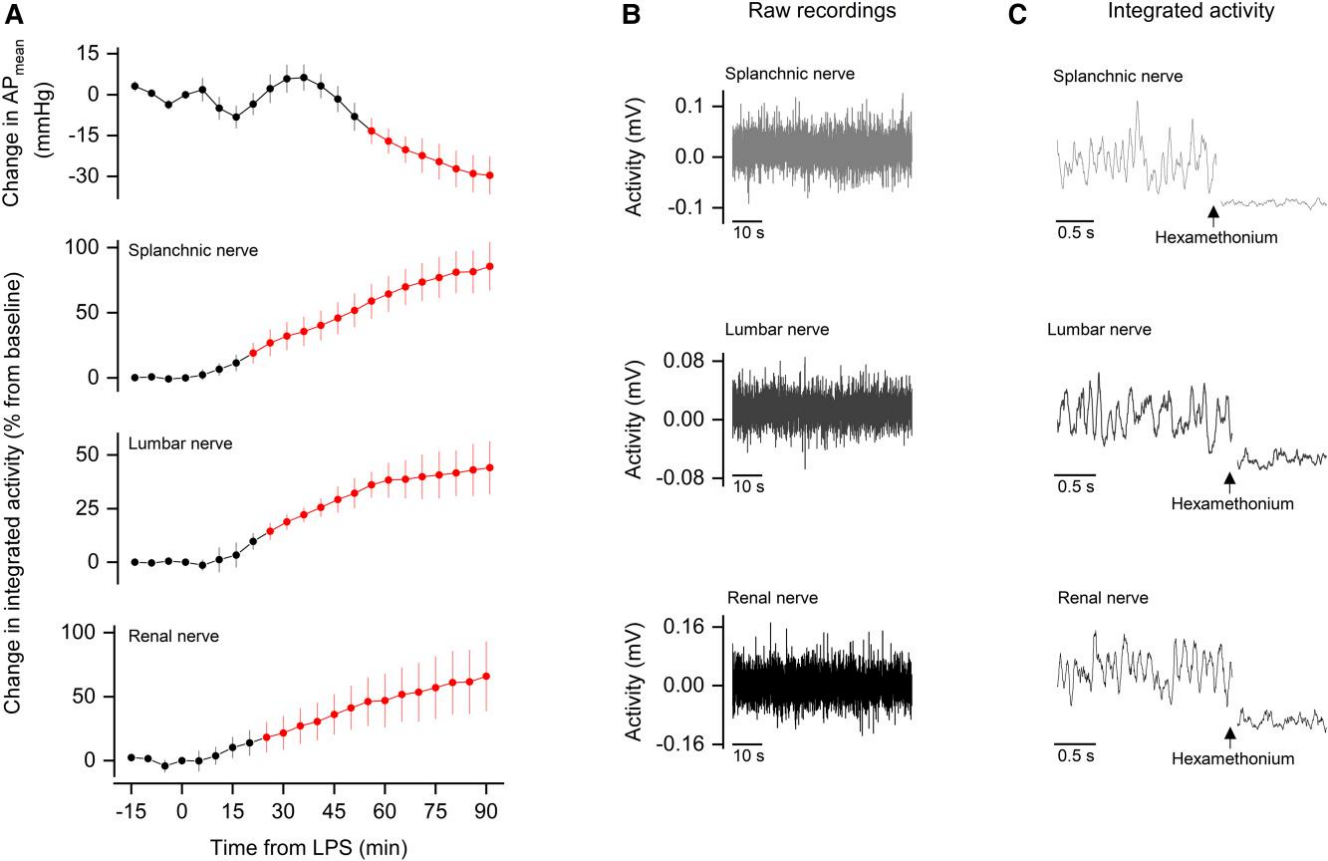
Sympathetic nerves that control cardiovascular function display enhanced activity during the early development of LPS-induced hypotension. (A) Time-dependent changes in sympathetic nerve activity, along with changes in AP_mean_, of rats challenged with LPS. Each nerve was recorded from six rats, not always simultaneously. Data are expressed as mean ± SEM. The red symbols and lines denote statistical difference in relation to the pre-LPS period. (B) Representative raw recordings of the splanchnic, lumbar, and renal nerves. (C) Effect of ganglionic blockade with hexamethonium on the integrated activity of the splanchnic, lumbar, and renal nerves.

Compared with the AP response of the unanesthetized rats, it took longer for the anesthetized rats to develop hypotension, with AP falling significantly below baseline at 55 min post-LPS (Fig. 3A), as opposed to 30 min post-LPS in the unanesthetized rats (Fig. 1B). The hypotensive response of the anesthetized rats was also somewhat less pronounced (by ∼10 mmHg) and was not accompanied by marked tachycardia (Supplementary Material Appendix, Fig. S4). Despite these differences, the fact that the anesthetized rats developed hypotension at a relatively early time point after the LPS challenge allowed for comparisons with the results obtained in the unanesthetized rats.

Representative nerve recordings from the anesthetized rats are shown in Fig. 3B. The recordings were viable for a sufficient amount of time to monitor the nerves until 90 min post-LPS. At the end of each experiment, sympathetic nerve activity was confirmed by ganglionic blockade with hexamethonium (Fig. 3C). These experiments revealed that integrated electrical activity did not decrease in any of the sympathetic nerves during the development of hypotension. In contrast, increases in nerve activity were observed even before the onset of hypotension, and such increases became progressively more pronounced as hypotension developed (Fig. 3A). Relative to baseline, the increase in nerve activity reached 86% for the splanchnic nerve, 44% for the lumbar nerve, and 66% for the renal nerve.

### Inactivation of the renin-angiotensin system does not take place at the onset of LPS-induced hypotension

Besides being under neural control, vascular tone is under endocrine control by angiotensin II. Therefore, we asked whether the early fall in SVR might be linked to inactivation of the renin-angiotensin system. Angiotensin II and its precursor, angiotensin I, were quantified by liquid chromatography coupled with tandem mass spectrometry (LC-MS/MS) in blood samples harvested at 30 min post-LPS. The results obtained, however, provided no evidence of inactivation of the renin-angiotensin system (Supplementary Material Appendix, Fig. S5).

### Changes in blood viscosity do not pose as a putative explanation for the early fall in SVR

According to the Hagen–Poiseuille law, viscosity is a determinant of resistance in nonideal fluid dynamic systems. Hence, its impacts on cardiovascular function ought to be taken into consideration. If blood viscosity happened to be reduced after LPS, it could promote a fall in SVR even if the average diameter of resistance vessels remained unaltered. Therefore, it was important to compare the rheological behavior of blood collected 30 min after LPS with that of blood collected from animals not challenged with LPS. We observed an exponential decay in viscosity as shear stress increased (Supplementary Material Appendix, Fig. S6), which is consistent with the non-Newtonian, pseudoplastic behavior of blood (21, 22). Such behavior, however, was unaffected by the LPS challenge in the time window of interest (Supplementary Material Appendix, Fig. S6).

### Frequency-domain analyses suggest that autoregulation is heightened at the onset of LPS-induced hypotension

In the setting of unchanged blood viscosity, dilation of resistance vessels is the most likely explanation for the fall in SVR. By considering this argument together with the facts that the fall in SVR was associated neither with vasoplegia ex vivo nor with brain-driven sympathetic shutdown in vivo, we reasoned that we are dealing with a mechanism that is present only in vivo, which is independent of the central nervous system, and which can overcome the vasoconstrictor effect of adrenergic stimulation. The local regulation of blood flow (tissue autoregulation) meets these criteria. Known to maintain flow relatively constant in tissues over an operational range of pressure, autoregulation consists of mechanisms that adjust resistance in direct proportion to pressure (23, 24). This is the opposite of what happens with the neural mechanisms of AP regulation (25, 26), as schematically represented in Supplementary Material Appendix, Fig. S7. Therefore, it is not unreasonable to hypothesize that these distinct mechanisms of cardiovascular regulation can become antagonistic under competitive demands, which might be the case in the early phase of LPS-induced hypotension. Also, in the setting of such an antagonism, autoregulation is known to be capable of rendering arterioles less responsive to adrenergic agonists and other vasoconstrictors (27).

To probe the systemic impacts of changes in tissue autoregulation, we initially performed a linear regression analysis for studying the behavior of SBF in relation to spontaneous changes in AP. This analysis revealed that SBF remained fairly constant during phase 2 of the response to LPS (Supplementary Material Appendix, Fig. S8), i.e. the phase in which hypotension developed. This is a characteristic of tissue autoregulation (23, 24) and was not noticeable at the systemic level in the phases that preceded or followed the hypotension (Supplementary Material Appendix, Fig. S8).

**Fig. 4.**
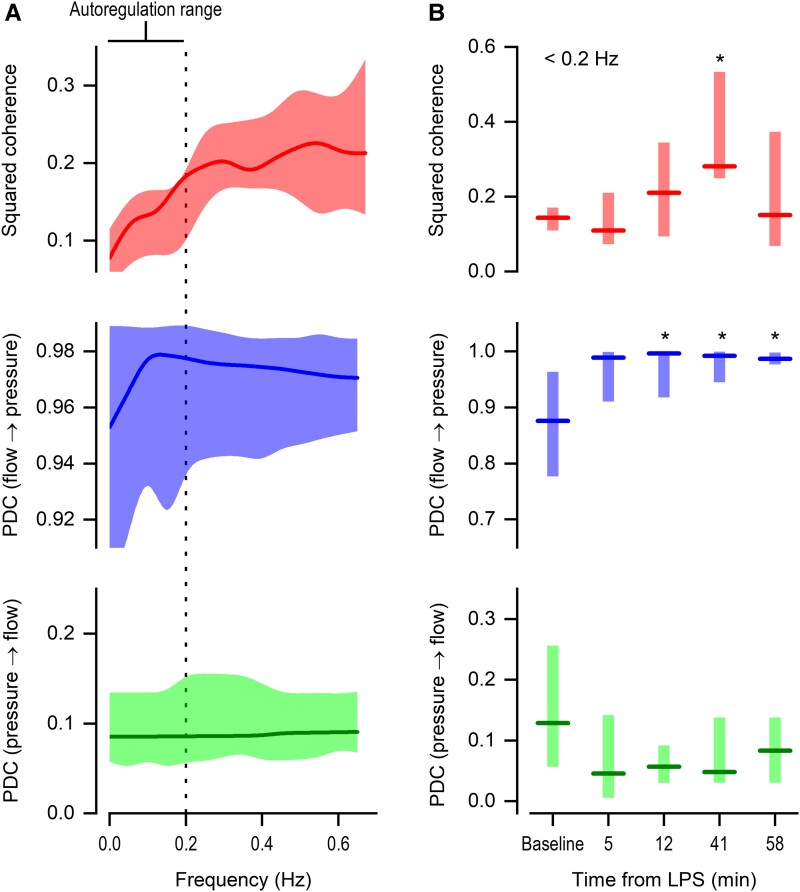
Frequency-domain proxies of autoregulation are heightened at the onset of LPS-induced hypotension. (A) Squared coherence and partial-directed coherence (PDC) as a function of frequency in control rats (not challenged with LPS). The low-frequency range known to be associated with the autoregulation of blood flow is indicated. (B) Effect of LPS on the low-frequency component of squared coherence and PDC (flow → pressure; and pressure → flow). The data are plotted as median (line) and 95% confidence interval (shaded area in A; floating bar in B); *n* = 9 rats. *Statistically different from the baseline period.

Next, we employed coherence analysis of spontaneous fluctuations in SBF and AP, extensively used to assess the strength of autoregulation at frequencies below 0.2 Hz (28, 29). The analysis revealed that, in the absence of the LPS, the SBF–AP coherence was relatively weak at less than 0.2 Hz (Fig. 4A). However, this proxy of autoregulation gained strength at the time corresponding to the onset of LPS-induced hypotension (41 min post-LPS; Fig. 4B).

We then used partial-directed coherence (PDC) to investigate the SBF–AP relationship. This is a frequency-domain counterpart of Granger causality and, as such, discriminates the SBF → AP relationship from the AP → SBF relationship (30). This analysis revealed that, even before the LPS challenge, the SBF → AP relationship was much stronger than the AP → SBF relationship at the frequency range in which autoregulation operates (<0.2 Hz; Fig. 4A). This makes sense if we consider that it is expected that the regulated parameter (SBF in the case of autoregulation) will be the one that drives adjustments in the other parameters in a closed-loop control system. The SBF → AP relationship in this frequency band strengthened after the challenge with LPS, reaching statistical difference from baseline already during the first hemodynamic phase (12 min), then being strengthened further during the second phase (41 and 58 min), to the point of becoming narrowly packed at the highest possible value (Fig. 4B). On the other hand, the frequency-matched AP → SBF relationship was not statistically affected during the response to LPS and even tended to get weakened (Fig. 4B).

### Autoregulatory escape is heightened during the onset of LPS-induced hypotension

**Fig. 5.**
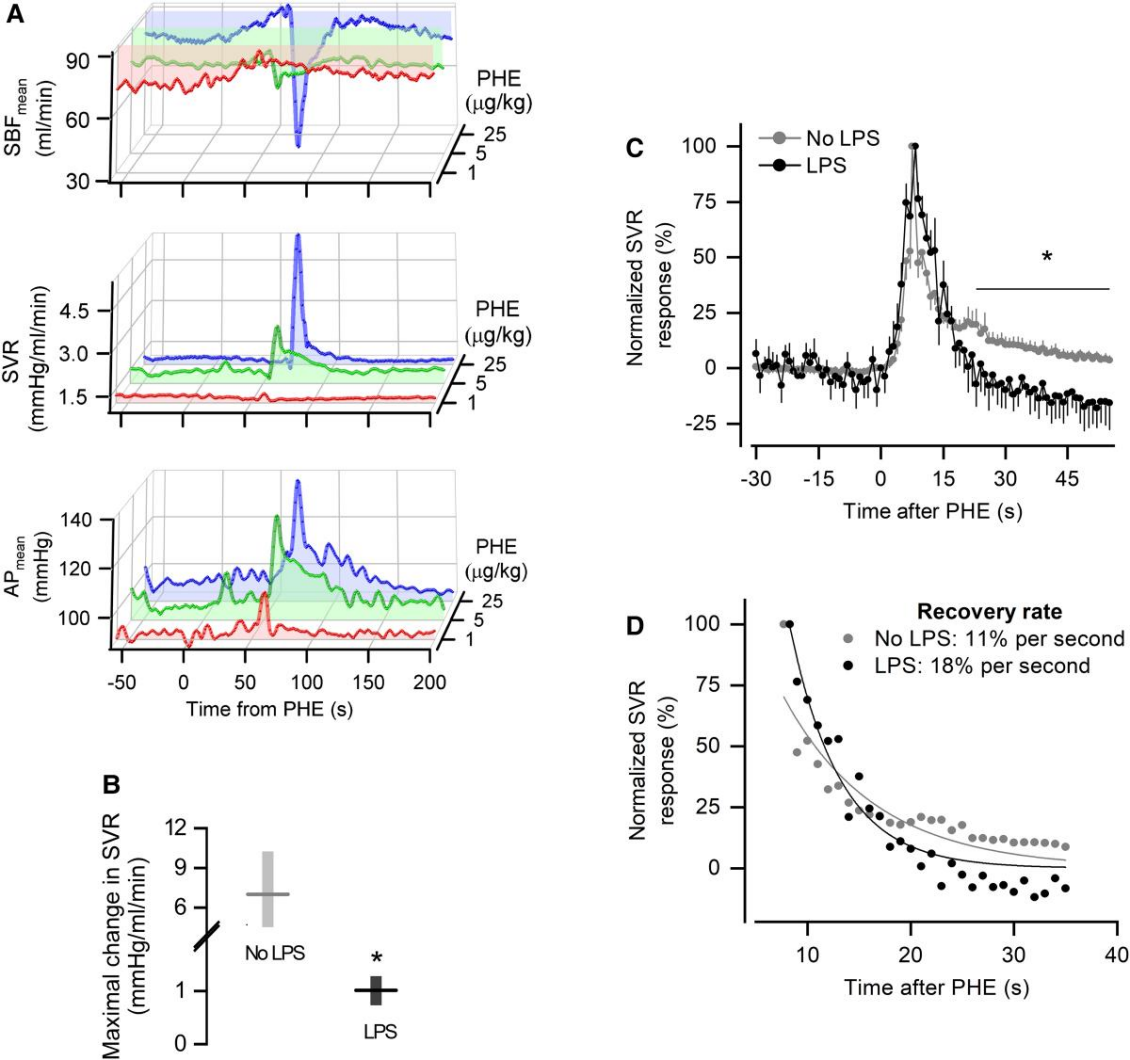
Autoregulatory escape is heightened during the onset of LPS-induced hypotension. (A) Characterization of the autoregulatory escape to PHE as transient changes in SBF_mean_, SVR, and AP_mean_. The doses of PHE tested are indicated; the 25-µg/kg dose was chosen for the subsequent experiments. (B) Magnitude of the rise in SVR induced by PHE given in the absence of LPS or at 40 min post-LPS. The data are expressed as median (horizontal line) and 95% confidence interval (floating bar). *Statistically significant effect of LPS. (C) Normalized SVR responses to PHE (peak at 100%) in the absence of LPS or at 40 min post-LPS. The data are expressed as mean ± SEM. This analysis was employed to evaluate whether LPS influenced the extent of the recovery (escape) from PHE-induced vasoconstriction. *Statistically significant effect of LPS. (D) Rate of recovery (escape) from PHE-induced vasoconstriction, as determined by exponential fitting of group means. In B–D, the group that received PHE in the absence of LPS consisted of 13 rats, whereas the group that received PHE after LPS consisted of 6 rats.

Seeking further evidence for altered autoregulatory control during the onset of LPS-induced hypotension, we assessed the phenomenon known as “autoregulatory escape” (31, 32). It refers to the recovery of resistance during continued vasoconstrictor stimulation, which, in the case of the present study, was achieved by an i.v. bolus injection of PHE (α_1_-adrenergic agonist). Despite the fact that PHE has an elimination half-life of 2–3 h, the hemodynamic responses caused by it lasted only for a few tens of seconds (Fig. 5A). As expected, the response consisted of an increase in SVR and a drop in SBF. A known aspect of this response is that although the baroreflex involved in AP regulation can modulate SBF during the response to PHE, it does not impact the rise in SVR or its recovery (31), presumably because PHE occupies virtually all α_1_ receptors and makes them unavailable for baroreflex-dependent sympathetic modulation. Accordingly, we found no correlation between PHE-induced bradycardia (a proxy of baroreflex) and the time for recovery in SVR (Supplementary Material Appendix, Fig. S9).

We then checked whether and how autoregulatory escape was altered at the time corresponding to the onset of LPS-induced hypotension. Contrary to what we observed in isolated resistance arteries (Fig. 2), the peak vasoconstrictor response to PHE was attenuated in vivo at the onset of LPS-induced hypotension, when compared with the group that received PHE in the absence of LPS (Fig. 5B). Although attenuation of the response peak might be related to heightened autoregulation, it is the recovery from vasoconstriction (autoregulatory escape itself) that is known to be a proxy of autoregulation (31, 32). To evaluate the rate of recovery from vasoconstriction, it was necessary to normalize (to 100%) the peaks of the SVR responses to PHE for both groups. When this was done, it became evident that the recovery was more complete in the group that received PHE at the onset of LPS-induced hypotension (Fig. 5C). This group also had a higher rate of recovery (Fig. 5D). These results are indicative of heightened autoregulatory escape during the development of hypotension.

### Hypovolemia drives the heightened autoregulation and the fall in SVR during the early hypotensive phase of systemic inflammation

**Fig. 6.**
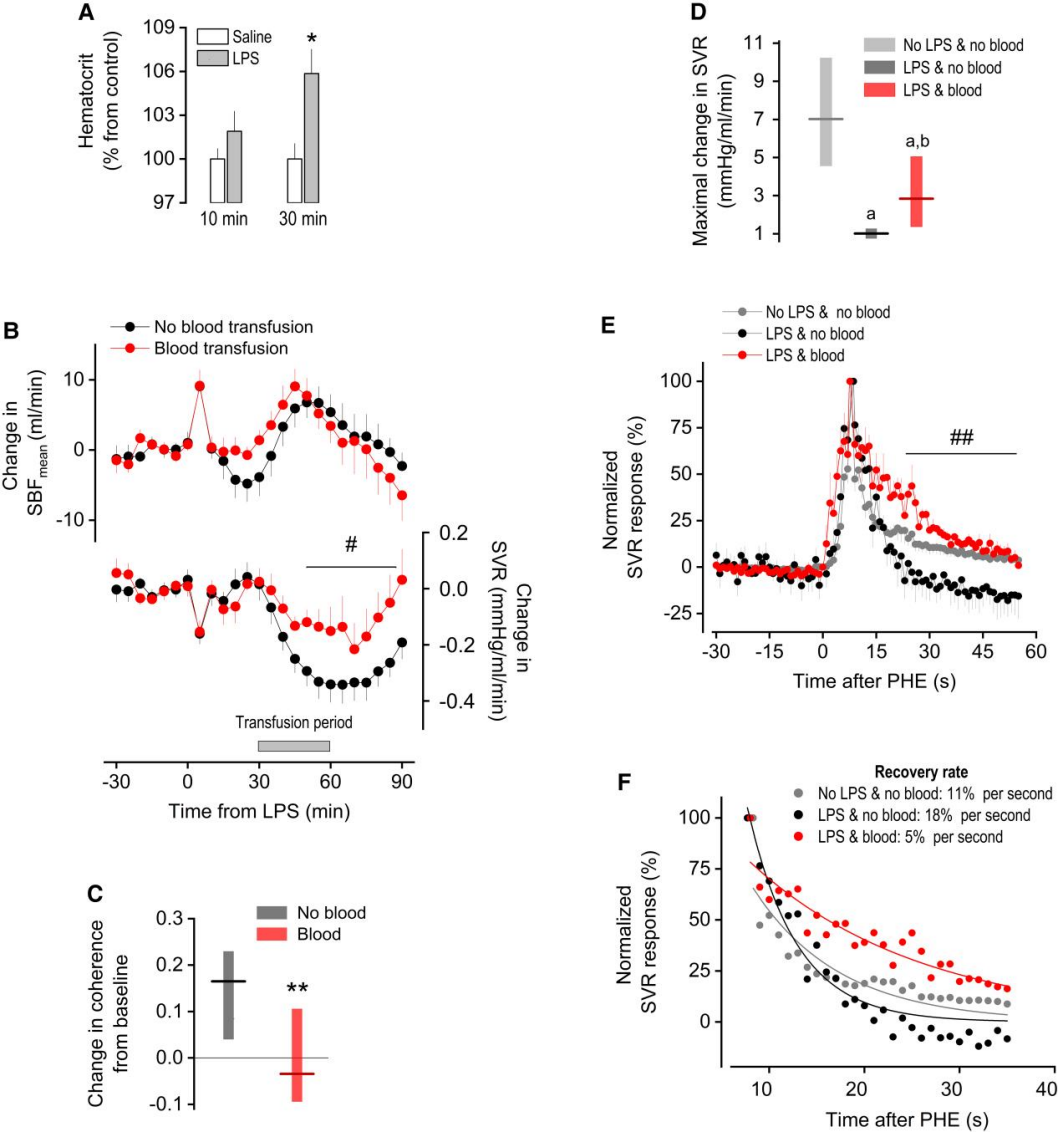
Hypovolemia as the putative cause of heightened autoregulation in the early phase of LPS-induced hypotension. (A) Hematocrit assessed at 10 or 30 min after the injection of LPS or saline. The data are expressed as mean ± SEM; *n* = 7–14 rats/group. *Statistical difference between the LPS and the saline groups. (B) Time course of the LPS-induced changes in SBF_mean_ and SVR in rats that received or did not receive blood transfusion. Transfusion began at 30 min post-LPS, as indicated. The data are expressed as mean ± SEM; *n* = 7–8 rats/group. ^#^Statistical difference between the groups that received and did not receive transfusion. (C) Low-frequency (<0.2 Hz) coherence at 60–70 min post-LPS in rats that received or did not receive blood transfusion. The data are expressed as change from baseline. Median (line) and 95% confidence interval (floating bar) are shown; *n* = 5 rats/group. **Statistically significant effect of blood transfusion. (D) Magnitude of the SVR rise induced by PHE in LPS-injected rats that received blood transfusion. The blood transfusion began at 30 min post-LPS, and PHE was bolus-injected at 40 min post-LPS. The data are expressed as median (horizontal line) and 95% confidence interval (floating bar); *n* = 5. For comparison, the effects of PHE in LPS-challenged rats that did not receive blood transfusion or in rats that received neither LPS nor blood transfusion are replotted from Fig. 5. ^a^Statistically different from the “no LPS & no blood” group. ^b^Statistically different from the “LPS & no blood” group. (E) Normalized SVR responses to PHE (peak at 100%) in LPS-injected rats that received blood transfusion. The data are expressed as mean ± SEM; *n* = 5. This analysis was employed to evaluate the extent of the recovery (escape) from PHE-induced vasoconstriction. For comparison, the effects of PHE in LPS-challenged rats that did not receive blood transfusion or in rats that received neither LPS nor blood transfusion are replotted from Fig. 5. ^##^Statistically significant effect of blood transfusion, compared with the LPS-injected rats that did not receive the transfusion. (F) Rate of recovery (escape) from PHE-induced vasoconstriction, as determined by an exponential fitting of group means. Group sizes as in D and E.

We hypothesized that fluid extravasation to tissues, with consequent hypovolemia, is the competitive demand that drives the prioritization of flow regulation over pressure regulation in the early hypotensive phase of systemic inflammation. Such extravasation is known to occur in the early stage of systemic inflammation as a result of an increased permeability of capillaries to proteins (12, 16), but whether the timing of this phenomenon matches that of the hypotensive response to LPS is not clear. Here, we used hemoconcentration (increase in hematocrit) as an index of fluid extravasation. At 10 min post-LPS (before the onset of hypotension), hematocrit did not differ from that of the time-matched, saline-injected controls (Fig. 6A). However, at 30 min post-LPS (onset of hypotension), a significant increase in hematocrit was observed (Fig. 6A).

Autologous blood transfusion was then employed to revert the hypovolemia. Because our goal was to revert hypovolemia without promoting a hypervolemic state, the rate of blood infusion was titrated so that it did not induce any sign of volume expansion, such as a rise in SBF above baseline (Fig. 6B) or reflexive bradycardia (Supplementary Material Appendix, Fig. S10). This was achieved with infusions performed at rates of 0.6 mL/min or less between 30 and 60 min post-LPS. This blood transfusion protocol significantly attenuated the fall in SVR induced by LPS (Fig. 6B). Such an effect is the opposite of what would be expected if SVR was being affected by the baroreflex response to volume expansion—baroreflex activation promotes a fall in SVR. We thus reasoned that, by exclusion, the effect that blood transfusion exerted on SVR in this experiment was likely to be related to the mechanisms of flow regulation.

To assess this matter more directly, two proxies of tissue autoregulation were evaluated in this experiment: coherence of spontaneous fluctuations in SBF and AP at frequencies <0.2 Hz; and the autoregulatory escape to PHE-induced vasoconstriction. Coherence was significantly reduced by blood transfusion in a time window (60–70 min post-LPS) that corresponded to the development of hypotension (Fig. 6C). Furthermore, coherence values did not differ from baseline in the blood-transfused, LPS-challenged rats (Fig. 6C), thus indicating that autoregulatory control was not strengthened in this experimental group. Regarding autoregulatory escape, we observed that blood transfusion restored, at least in part, the peak response to i.v. PHE at 40 min post-LPS (Fig. 6D). The response peaks were then normalized to 100% in all groups, so that completeness of the recovery (Fig. 6E) and recovery rate (Fig. 6F) could be evaluated. These indices of autoregulatory escape were both affected by the blood transfusion in a way that was consistent with a reversion of the LPS-induced effects.

## Discussion

Previous studies sought to investigate whether the local regulation of flow (tissue autoregulation) could become impaired in clinical sepsis and in the LPS model of systemic inflammation, focusing on the putative link between such an impairment and organ dysfunction. But while these studies found evidence of impaired autoregulation in advanced stages of such conditions (33–35), they did not find evidence of autoregulation dysfunction in earlier stages (36–40). Some of the previous studies even found evidence of an enhanced autoregulatory gain in the early stage of LPS-induced systemic inflammation (34, 38–40). The present study now adds a new facet to the relationship between autoregulation and systemic inflammation by providing evidence that such local mechanisms can sum up to exert systemic effects and cause the early development of hypotension. More specifically, in order to maintain blood flow during the early development of hypovolemia in the LPS-challenged rats, autoregulation was heightened and drove both resistance and pressure downward. The effectiveness of this compensatory mechanism seems to rely on the ability of autoregulation to render blood vessels irresponsive to sympathetic nerves. Accordingly, a seminal study (27) performed in dogs has shown that physiologically relevant decreases in the perfusion pressure of an autoregulating kidney render the arterioles of this organ less responsive not only to norepinephrine, but also to epinephrine and angiotensin II.

The mechanisms of early hypotension uncovered herein are quite distinct from the mechanism known to bring about hypotension in the later stages of systemic inflammation, when injury to blood vessels impairs their intrinsic contractile properties, leading to a state of uncontrolled vasodilation, referred to as vasoplegia (8–10). The few studies that have evaluated vasoreactivity to adrenergics within the first hour of an LPS injection have observed an attenuation of vasopressor responses to norepinephrine and PHE in vivo and interpreted these findings as evidence of vasoplegia (41, 42). The present study now suggests that such an early in-vivo attenuation is more likely to result from heightened autoregulation. We argue that ex-vivo experiments are better suited to probe for vasoplegia, because the parenchyma-derived metabolic signals that drive autoregulation are absent in isolated arteries. In the present study, we used an ex-vivo preparation of third-order, resistance mesenteric arteries and found no evidence of vascular dysfunction in the early stage of LPS-induced hypotension. To our knowledge, no other study has evaluated the behavior of resistance arteries harvested within the first hour of an LPS challenge. Also, although there are a couple of studies (43, 44) that evaluated isolated aortic rings and found a certain level of dysfunction, it should be considered that the aorta is a capacitance artery rather than a resistance artery, thus contributing very little to SVR.

From an evolutionary perspective (45), it is presumable that an addition of the neuroendocrine mechanisms of pressure regulation on top of the local mechanisms of flow regulation, or vice versa, has provided adaptive advantages. Otherwise, it is unlikely that these two independent control systems would coexist in the most derived species. However, this does not imply that the local regulation of flow and the brain-driven regulation of pressure always cooperate with each other. Antagonistic actions between these two control systems can be expected based on the fact that they exert opposing effects on vascular resistance: in the flow autoregulation, increases in pressure are met with increases in vascular resistance; in the regulation of pressure (baroreflex), increases in pressure are met with decreases in resistance (Supplementary Material Appendix, Fig. S8). According to the present study, the early hypotensive phase of LPS-induced systemic inflammation appears to be a situation in which these two control systems become antagonistic. In this particular case, the local autoregulatory mechanisms override the neuroendocrine mechanisms so that SBF could be maintained at the expense of a reduced AP.

Considering that flow autoregulation is a local mechanism, it will be necessary for future studies to assess the relative contribution of each organ to the systemic manifestation of autoregulation that takes place during the early stage of LPS-induced hypotension. Under resting conditions in healthy subjects, it is known that autoregulation operates with the highest gain in the brain, heart, and kidneys, with a moderate gain in the skeletal muscle, and with a low gain in the liver, gut, and stomach (23). However, shortly after an LPS challenge, autoregulation could be strengthened not only in the organs in which it is strong to begin with, but probably even more so in the organs with weaker baseline autoregulation. In line with this argument are studies showing that blood flow in the skeletal muscle (46, 47), stomach (48, 49), and gut (50) becomes more dependent on autoregulation as part of physiological adaptations to certain conditions such as exercise and feeding. A caveat to consider, though, is that increased metabolic activity is thought to be an important drive for heightened autoregulatory gain in these particular adaptations (46–50), which does not correspond to what occurs in the early stage of LPS-induced hypotension—a regulated suppression in the whole-body metabolic rate occurs instead (51). But the metabolic rate is not the only mechanism capable of strengthening autoregulation. For example, the P450-derived eicosanoid 20-HETE and the superoxide anion have been shown to strengthen autoregulation, at least in the kidney (52). Hence, it is reasonable to suggest that autoregulation in LPS-induced hypotension might be modulated by inflammatory mediators and oxidative stress, presumably combined with the hypovolemia-related signals.

Looking ahead, much research is still needed to assess whether the hypothesis raised in the present study can be of clinical significance. The next logical step is to test the hypothesis in animal models of systemic inflammation induced by living bacteria, among which cecal ligation and puncture (CLP) is the most commonly used. Hemodynamic alterations in the CLP model have yet to be studied with high temporal resolution, but the available data suggest the existence of at least two phases: an earlier phase at 5–6 h in which hypotension is associated with increased blood flow and a later phase at 20–24 h in which hypotension is associated with decreased blood flow (53, 54). It is thus reasonable to speculate that, similarly to what we have observed in the LPS model, the autoregulation of blood flow might take priority over pressure regulation early in the CLP model, having as a consequence the development of hypotension. Another point that warrants future investigation is whether there is a mechanistic link between the contribution of autoregulation to the development of hypotension and the therapeutic effectiveness of fluid therapy in septic shock. As pointed out in the Introduction, only 50% of the septic-shock patients are responsive to crystalloid fluids infused to support volemia (6, 7), but the mechanism underlying this fact is unclear. Based on the results of the present study, it is reasonable to propose that fluid therapy may be effective only when heightened autoregulation is the mechanism driving the development of hypotension. Testing this possibility in animal models of bacterial sepsis, as well as in septic patients, might be a way to reach a mechanism-oriented approach to fluid therapy.

### Conclusion

By tracking systemic hemodynamics with the highest temporal resolution in freely moving, unanesthetized rats, the present study uncovered dynamic transitions and provided novel mechanistic insights regarding the early stage of LPS-induced systemic inflammation. Most notable are the findings leading to the hypothesis that heightened gain in the autoregulation of blood flow drives the early development of hypotension. This working hypothesis provides a new paradigm for scientific investigation.

## Methods

### Animals and surgical preparation

The study was conducted in male Wistar rats obtained from the specific pathogen-free facility of the University of Sao Paulo. All protocols were approved by the Animal Care and Use Committee at the Institute of Biomedical Sciences of the University of Sao Paulo. One week before an experiment, the rats were subjected, as necessary, to one or more of the following surgical procedures: implantation of a transit-time flow probe around the ascending aorta; implantation of a pressure telemetric sensor in the abdominal aorta; and implantation of an i.v. (jugular) catheter. Surgeries were performed aseptically under anesthesia with isoflurane (1.5–2.5%), as detailed in Supplementary Methods.

### In-vivo experimental setup

On the day of the experiment, unanesthetized, freely moving rats were transferred to an environmental chamber (Environmental Growth Chambers) set to 21°C, an ambient temperature that is preferred by rats in the early stage of LPS-induced hypotension (55). Each singly housed rat was equipped with an infusion harness, which protected a PE-50 extension of the i.v. catheter. An extension cable of the flow probe was connected to the implant that was fixed to the head of the animal. The cable and catheter extensions were passed by a swivel system. The cable extension was plugged into a TS420 perivascular flow module (Transonic System). The catheter extension was connected to a syringe located outside the environmental chamber, from where all injections and infusions were performed. A PhysiolTel RPC-1 receiver (Data Sciences) positioned under each cage captured the radio waves emitted by the telemetry transmitter. The signals captured by the flow module and the telemetry receiver were conveyed to a computer. The signals were recorded at 500 Hz by using the Data Sciences ART software.

### Ex-vivo assessment of vascular reactivity

At the time points of interest, rats in the in-vivo experimental setup were anesthetized with an i.v. bolus injection of thiopental (20 mg/rat) and promptly transferred to the tissue harvesting tray. The mesenteric vascular bed was excised, and segments of third-order resistance arteries were evaluated for isometric force development, as previously described (56). For details, see Supplementary Methods.

### Extracellular nerve recordings

The electrical activities of the splanchnic, renal, and lumbar nerves were recorded from rats under isoflurane (1.5–2.5%) anesthesia using extracellular bipolar electrodes. The experimental preparation was similar to that described previously (57). Details are provided in Supplementary Methods.

### LC-MS/MS method of angiotensin quantification

Blood was sampled from the inferior vena cava immediately after rats in the in-vivo experimental setup were anesthetized with i.v. thiopental (20 mg/rat). The blood was mixed with the following cocktail of anticoagulant and protease inhibitors (final concentrations): *p*-hydroxymercuribenzoate (5 mM); phenylmethylsulfonyl fluoride (10 µM); EDTA (7.5%); *o*-phenanthroline (150 mM); and pepstatin (2 mM). The plasma was obtained by centrifugation (3,000 × *g*, 10 min, 4°C). The plasma samples were then subjected to solid-phase extraction and LC-MS/MS as previously described (58, 59).

### Determination of blood viscosity

Blood samples were harvested as for the angiotensin assay, except that heparin (instead of the cocktail) was used for sample preparation. The rheological behavior of the blood samples was evaluated using an R/S Plus controlled stress rheometer (Brookfield Engineering Laboratories) equipped with an RCT-75-1 cone and a bath circulator for temperature control (37°C). The analyses were performed under shear stress control conditions, ramping it up linearly from 0 to 15 Pa in 100 s.

### Hematocrit

Blood samples were harvested as for the angiotensin assay, except that EDTA was used for sample preparation. The sample was analyzed on a fully automated hemocytometer (Mindray BC-2800Vet, Shenzhen Mindray Bio-Medical Electronics).

### Drugs

LPS from *Escherichia coli* O127:B8, PHE, KCl, serotonin, acetylcholine, and hexamethonium were obtained from Sigma-Aldrich. LPS was suspended in saline and sonicated in bath for 30 min immediately before use. The other drugs were dissolved in saline without the need for sonication. In the in-vivo experiments, LPS (1 mg/mL), PHE (1–25 µg/mL), or hexamethonium (30 mg/mL) were bolus-injected i.v. at a volume of 1 mL/kg. In the ex-vivo vascular reactivity experiment, a concentrated solution of PHE, KCl, serotonin, or acetylcholine was added to the myograph chamber in a volume that did not exceed 4% of the end chamber volume.

### Autologous blood transfusion

One week before the surgical preparation, blood (3.0 mL) was collected directly from the jugular vein, under isoflurane anesthesia. The blood was mixed with a blood-bank preserving solution and stored at 4°C. On the day of the experiment, the blood was warmed to 37°C, and the autologous transfusion was performed via the extension of the venous catheter. The transfusion was made at a rate of 0.1 mL/min for 30 min in the experiments aimed at evaluating minute-to-minute changes induced by LPS. Also, it was made at a rate of 0.6 mL/min for 5 min in the experiments aimed at evaluating the more rapid changes induced by PHE. In either case, the transfusion began at 30 min post-LPS. Control rats did not receive the blood transfusion, but had their blood collected in the same way as the rats designated to receive the transfusion.

### Data processing and analyses

See Supplementary Methods for information on data processing, as well as on the principles and equations of the coherence and PDC analyses. The codes used in the coherence and PDC analyses have been made freely available in the GitHub code hosting platform (https://github.com/abnr/hemodynamic). Statistical comparisons were performed using Statistica 8.0 or SAS Enterprise 9.2, with the level of significance set to 0.05. The following parameters were not normally distributed: coherence, PDC, EC_50_ for ex-vivo vascular contraction, maximal ex-vivo contraction, and peak SVR responses to i.v. PHE. All other parameters were normally distributed. The non-normally distributed data were expressed as median and 95% confidence interval (determined by bootstrapping). The normally distributed data were expressed as mean ± SEM. Nonparametric statistics were used to evaluate the parameters that were not normally distributed: the Mann–Whitney test was used for pairwise comparisons; the Kruskal–Wallis test was used for multiple comparisons. Time-dependent changes in the normally distributed parameters were evaluated using a linear mixed-effects model, with treatment set as the fixed effect, time as the repeated measurements, and subjects as the random effect. Atemporal comparisons of the normally distributed parameters were performed by using the t test (2 groups) or ANOVA (≥3 groups). The ANOVA was followed by the Fisher's least significant difference test.

## Supplementary Material

pgad014_Supplementary_Data

## Data Availability

All data needed to evaluate the study are included in the paper or the Supplementary materials. The codes used in the coherence and PDC analyses have been made freely available in the GitHub code hosting platform (https://github.com/abnr/hemodynamic).
