# Supplementary material for: Autoregulation of blood flow drives early hypotension in a rat model of systemic inflammation induced by bacterial lipopolysaccharide
Source: PNAS Nexus. 2023 Jan 21;2(2):pgad014. doi: 10.1093/pnasnexus/pgad014 (PMC9982072; doi:10.1093/pnasnexus/pgad014)
Supplement: pgad014_Supplementary_Data [file pgad014_supplementary_data.zip › PNASNEXUS-PNASNEXUS-2022-00696-T-s01.pdf]

SUPPLEMENTARY METHODS

Autoregulation of blood flow drives early hypotension in a rat model of systemic inflammation induced by bacterial lipopolysaccharide.

Moretti EH, Rodrigues AC, Marques BV, Totola LT, Ferreira CB, Brito CF, Matos CM, Silva FA, Santos RAS, Lopes LB, Moreira TS, Akamine EH, Baccala LA, Fujita A, Steiner AA.

Surgical procedures. Surgeries were performed aseptically under anesthesia with isoflurane (1.5-2.5%). Body temperature (monitored by a colonic thermocouple) was maintained between 36.5ºC and 37.0ºC with the help of an isothermal pad. The rats were provided with antibiotic prophylaxis (enrofloxacin, 5 mg/kg s.c.) prior to surgery, and initiated on a 3-day pain management protocol (ketoprofen, 5 mg/kg s.c., 1-2 doses/day) at the end of surgery.

For implantation of the flow probe, the rats were mechanically ventilated (4 ml/breath; 60-70 breaths/min) through an endotracheal tube. After the thoracic cavity was accessed by a median sternotomy, the aortic arch was isolated and cleared from connective tissue by blunt dissection. An ultrasonic transit-time flow probe (model MC2.5PSB; Transonic Systems, Ithaca, NY, USA) was then fitted around the ascending aorta, with its J-reflector facing away from the pulmonary artery. The probe's cable was passed through the sternal incision and tunneled under the skin to the nape. Its four-pin connector was fixed to the skull with the help of a rigid cuff, micro screws and acrylic cement. The sternum was closed with stainless steel wires and the chest's negative pressure was restored through an inter-rib pinhole sealed with a purse-string suture. The incision sites in muscle and skin were then sutured.

The telemetry transmitter (model HD-S10; Data Sciences International, St. Paul, MN, USA) consisted of a capsule joined to a gel-filled catheter. A midline laparotomy was performed for its implantation. The abdominal aorta was accessed by gently displacing the

small intestine sideways with saline-soaked gauze pads. The aorta was temporarily ligated caudal to the renal arteries while the catheter was being inserted and secured in place with octyl-cyanoacrylate glue. Blood flow was restored within 2 min. The transmitter capsule was sutured to the ventral muscles during the closing process. The incision site in skin was then sutured.

For the venous catheterization, the left external jugular was isolated and permanently ligated. A 3-Fr polyurethane catheter was inserted into the vein caudally to the ligation site, and then advanced until its tip reached the right atrium. The distal end of the catheter was passed under the skin and exteriorized at the nape, after which the catheter was locked with heparinized glycerol (500 U/ml).

***Ex-vivo* assessment of vascular reactivity.** Artery segments of 2 mm in length were mounted on a wire myograph (Danish Myo Technology), and maintained at 37ºC in a modified Krebs-Henseleit solution aerated with 95% O_2 and 5% CO_2. The modified solution consisted of NaCl at 130 mM, $NaHCO_3$ at 14.9 mM, KCl at 4.7 mM, KH_2PO_4 at 1.2 mM, $MgSO_4$ at 1.2 mM, D-glucose at 5.5 mM, $CaCl_2$ at 1.6 mM and EDTA at 26 μM. The analog output of the wire myograph was digitally converted by Power Lab (AD Instruments), and then recorded in a computer with the help of the Lab Chart software. After the relationship between passive wall tension and internal circumference was traced, the internal circumference was set to the transmural pressure of 93 mmHg using a normalization module (Danish Myo Technology). Then, cumulative concentration-response curves to vasoactive agents were constructed. A 30-min wash out period with the modified Krebs-Henseleit solution was allowed between the tests with each vasoactive agent.

Extracellular nerve recordings. The electrical activities of the splanchnic, renal and lumbar nerves were recorded from rats under isoflurane (1.5-2.5%) anesthesia using extracellular bipolar electrodes. Prior to isolation of the target nerve fibers, all rats were subjected to the following procedures: carotid artery catheterization for arterial pressure (AP) measurement; femoral vein catheterization for drug administration; and intubation via tracheostomy for ventilatory support (4 ml/breath; 60-70 breaths/min). The respiratory flow and the expired fraction of CO_2 (3.5-4.5%) were monitored via a capnograph (Microcapstar-100; CWE) connected to the endotracheal tube. Body (colonic) temperature was maintained at 36.5-37.0ºC with the help of an isothermal pad.

Using a retroperitoneal approach, the left splanchnic nerve and the left renal nerve were isolated distally to their ganglia. The right lumbar nerves were accessed by a ventral midline laparotomy followed by displacement of the intestines, abdominal aorta and vena cava. Each nerve was then placed on a pair of Teflon-coated, stainless-steel electrodes with bared tips (A-M Systems). A silicone sealant was applied to fix and protect the nerve-electrode junctions. The surgical wounds were closed with suture. The raw signal was amplified 10,000 times (Model 1700 differential AC amplifier, A-M Systems), digitally converted (Micro 1401 Data Acquisition Unit, Cambridge Electronic Design), and acquired in a computer with the help of the Spike2 software (Cambridge Electronic Design). At the end of each experiment, sympathetic nerve activity was confirmed by ganglionic blockade with hexamethonium (30 mg/kg i.v.).

Data processing and analyses

Pulsatile AP and SBF data from unanesthetized rats were analyzed in Ponemah (Data Sciences International) to obtain minute-to-minute values of AP_{mean}, SBF_{mean}, heart rate, stroke volume and dP/dt_{max}. SVR was calculated as the ratio of AP_{mean} by SBF_{mean}. The first

derivatives of AP_{mean} and SVR were calculated in Origin Lab. Linear and exponential regressions were also performed in Origin Lab. In the evaluation of autoregulatory escape, SVR responses were normalized on a second-by-second basis using the following equation: $(SVR_t - SVR_i) / (SVR_{max} - SVR_i) \times 100\%$, where SVR_t is the value of SVR at each time point, SVR_i is the initial value of SVR averaged over the 30 s that preceded the injection of PHE, and SVR_{max} is the maximum value of SVR achieved during the response to PHE.

The time-series of AP and SBF were subjected to frequency-domain analyses of coherence and PDC (partial directed coherence). For the analyses, the series were separated in windows of 5 min, and filtered using a passband FIR filter from 0.01 to 1.2 Hz. Coherence is a nondirectional measure of phase synchronization defined as $C_{i,j}(f) = \frac{|S_{ij}(f)|^2}{S_{ii}(f)S_{jj}(f)}$, where $S_{ij}(f)$ is the cross-spectrum between two time series i and j for frequency f. PDC is a directional measure of phase synchronization that can be defined in multivariate autoregressive models (MVAR). Let $Y(k) = [y_1(k), \ldots y_N(k)]^T, 1 \leq k \leq n$ be an N-dimensional time series, p be the number of lagged observations of the model, $A \in R^{N \times N}$ be the matrix with the coefficients at lag r, and $E(n)$ be the noise matrix at point n. The N-dimensional time series is then modeled as an MVAR model: $Y(n) = \sum_{r=1}^{p} A_r Y(n-r) + E(n)$. Using Fourier methods, we transform the MVAR model to the frequency domain representation as $A(f) = I - \sum_{r=1}^{p} A_r e^{-i2\pi f}$, where I is the identity matrix. Finally, the PDC is defined as $PDC_{j \to i}(f) = |A_{ij}(f)| / \sqrt{\sum_{k=1}^{H} |A_{kj}(f)|^2}$ (*Biological Cybernetics* 84: 463-474, 2001). Both coherence and PDC measures lie in the interval [0,1]. The code was run on MATLAB; it has been made freely available in the GitHub code hosting platform (https://github.com/abnr/hemodynamic).

Electrical nerve activity was analyzed offline in the Spike2 software. The raw signals were filtered at 100-1,000 Hz, rectified, smoothed, and then integrated. The associated pressure

recording was analyzed unfiltered, and heart rate was derived from it. Integrated nerve activity, AP_{mean} and heart rate were computed at 5-min intervals for the duration of the experiment.

In the isometric contraction/dilation experiments, Power Lab was used to obtain measures of wall tension as force per unit of wall length. The relationship between resting tension and internal circumference was plotted against an isobar function (tension = transmural pressure x internal circumference / 2 π) corresponding to the transmural pressure of 93 mmHg. The internal circumference corresponding to the working transmural pressure was found at the intersection of the two curves. The obtained internal circumferences were then used in the isometric contraction/dilation experiments. Using Origin Lab, EC_{50} and response maximum were calculated for each vasoactive agent tested in the isometric contraction/dilation experiments.
