# Supplementary material for: Autoregulation of blood flow drives early hypotension in a rat model of systemic inflammation induced by bacterial lipopolysaccharide
Source: PNAS Nexus. 2023 Jan 21;2(2):pgad014. doi: 10.1093/pnasnexus/pgad014 (PMC9982072; doi:10.1093/pnasnexus/pgad014)
Supplement: pgad014_Supplementary_Data [file pgad014_supplementary_data.zip › PNASNEXUS-PNASNEXUS-2022-00696-T-s02.pdf]

SUPPLEMENTARY FIGURES

Autoregulation of blood flow drives early hypotension in a rat model of systemic inflammation induced by bacterial lipopolysaccharide. Moretti EH, Rodrigues AC, Marques BV, Totola LT, Ferreira CB, Brito CF, Matos CM, Silva FA, Santos RAS, Lopes LB, Moreira TS, Akamine EH, Baccala LA, Fujita A, Steiner AA.

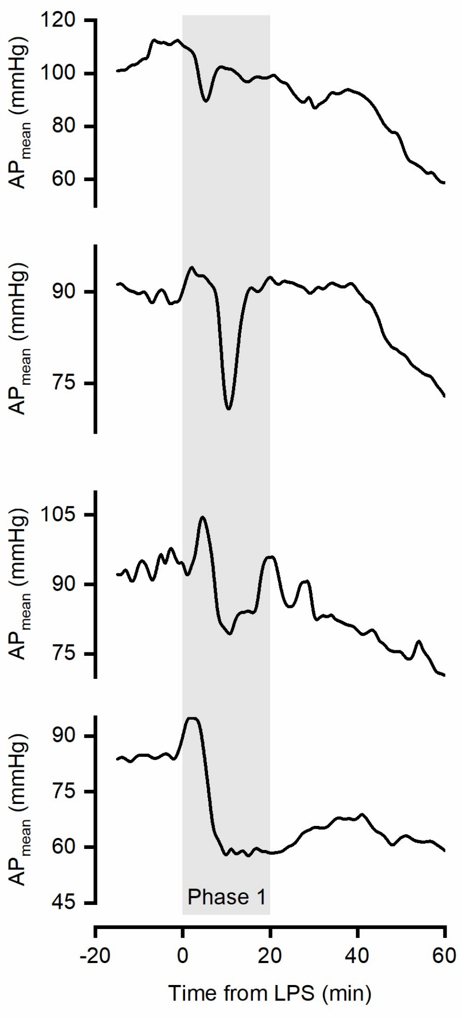

Figure S1. AP_{mean} recordings of the 4 of 11 individual rats that presented hypotension during the phase 1 of the response to LPS.

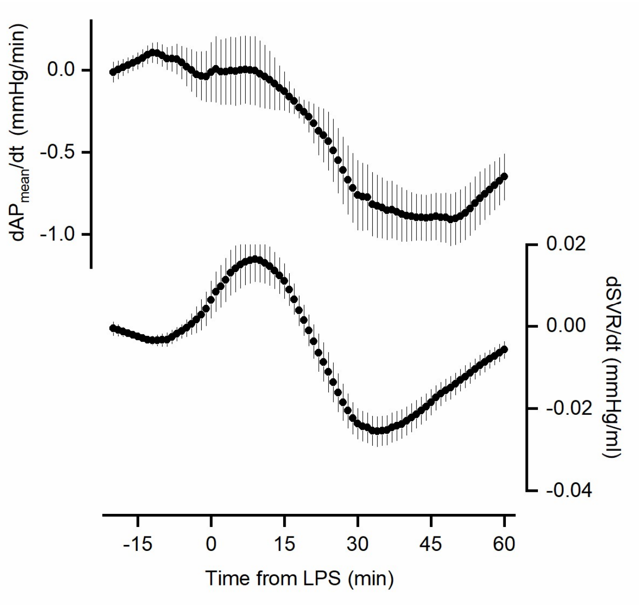

Figure S2. Derivatives of mean arterial pressure (dAP_{mean}/dt) and systemic vascular resistance (dSVR/dt) as a function of time in the LPS-challenged rats. Data are expressed as means ± SEM; n = 11 rats.

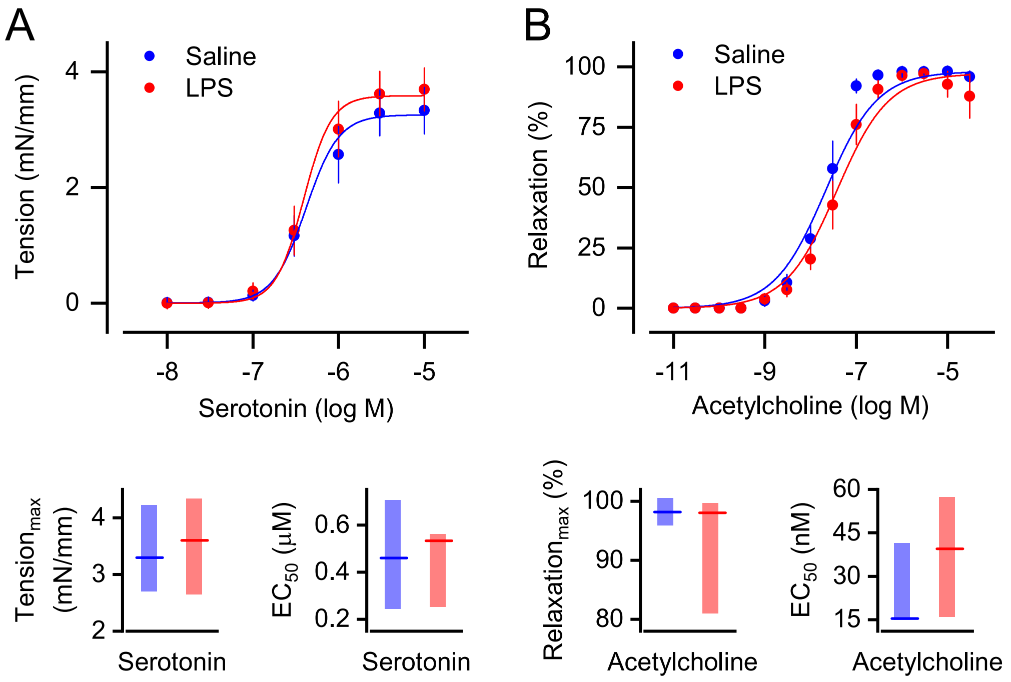

Figure S3. The intrinsic contractile properties of resistance mesenteric arteries are preserved at the time corresponding to the onset of hypotension (30 min post-LPS). (A) Concentration-dependent contractile responses to serotonin. (B) Concentration-dependent dilatory responses to acetylcholine in mesenteric arteries precontracted with PHE. In each panel are displayed the concentration-dependent tension curves, as well as the corresponding response maxima ($Tension_{max}$) and EC_{50}. Concentration-dependent curves are plotted as means ± SEM. $Tension_{max}$ and EC_{50} data often failed to meet a normal distribution and are expressed as median (horizontal line) and 95% confidence interval (floating bar). Five to eight arteries from different rats were tested with each vasoactive agent.

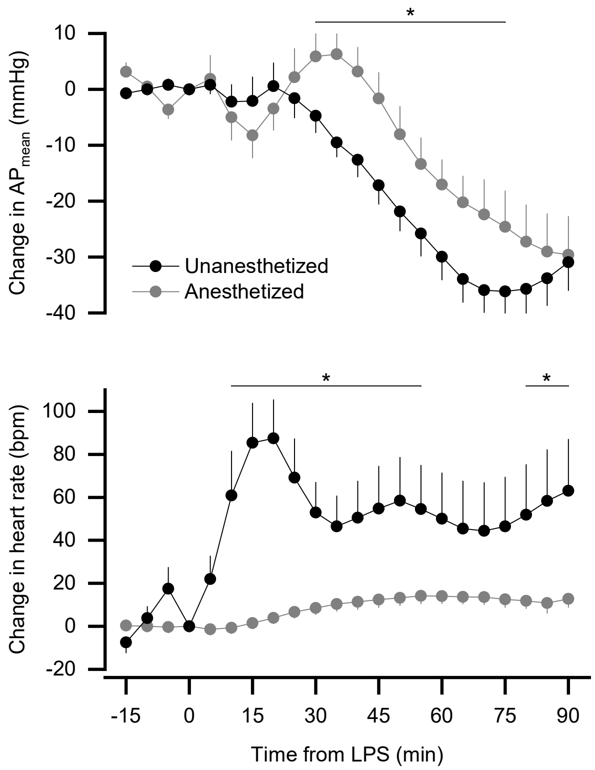

Figure S4. Comparison of AP_{mean} and heart rate responses to LPS in unanesthetized versus anesthetized rats. Data are expressed as means ± SEM; n = 10-11 rats. *Significant difference between unanesthetized and anesthetized rats.

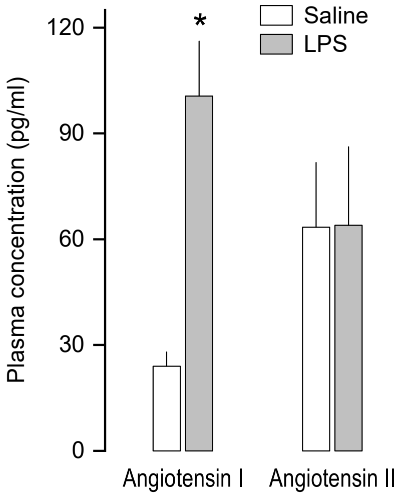

Figure S5. Status of the renin-angiotensin system at the onset of LPS-induced hypotension (30 min post-LPS) in comparison with time-matched saline-injected controls. The plasma concentrations of angiotensin I and II are shown as means ± SEM; n = 6-10 rats/group. *Statistically different from the saline-injected group.

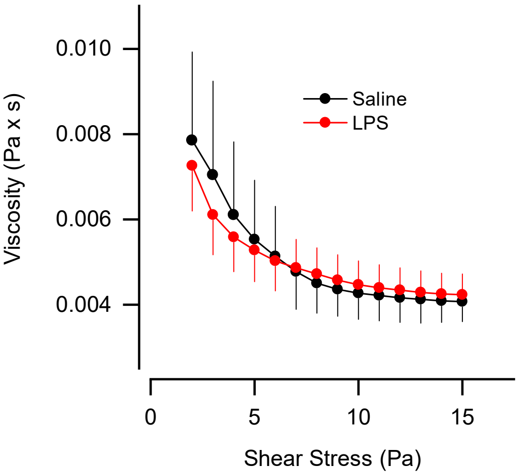

Figure S6. Rheological behavior of blood inferred from the relationship between viscosity and shear stress. Blood was collected 30 min after the i.v. injection of LPS or saline. Data are expressed as means ± SEM; n = 5-7 rats/group. There was no statistical difference between the groups.

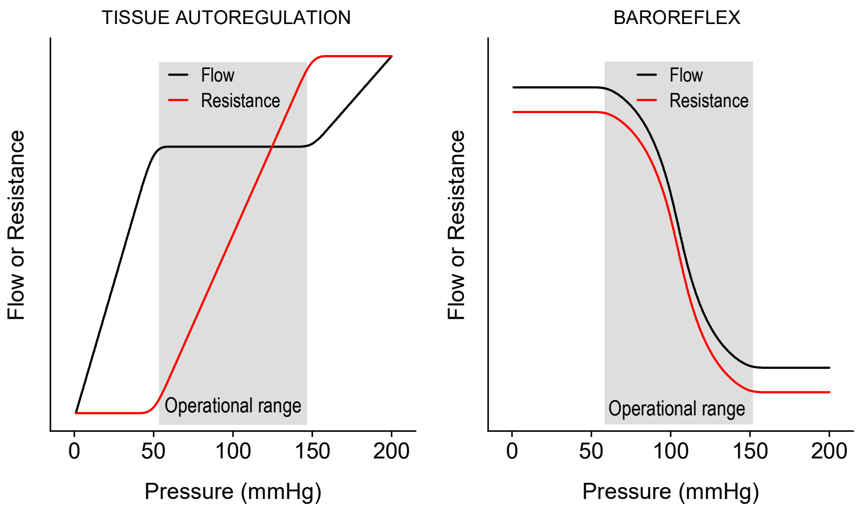

Figure S7. Known interplay among blood flow, resistance and pressure in tissue autoregulation (**A**) and in the neural regulation of arterial pressure (baroreflex, **B**). Based on data reviewed in Refs. 23-26.

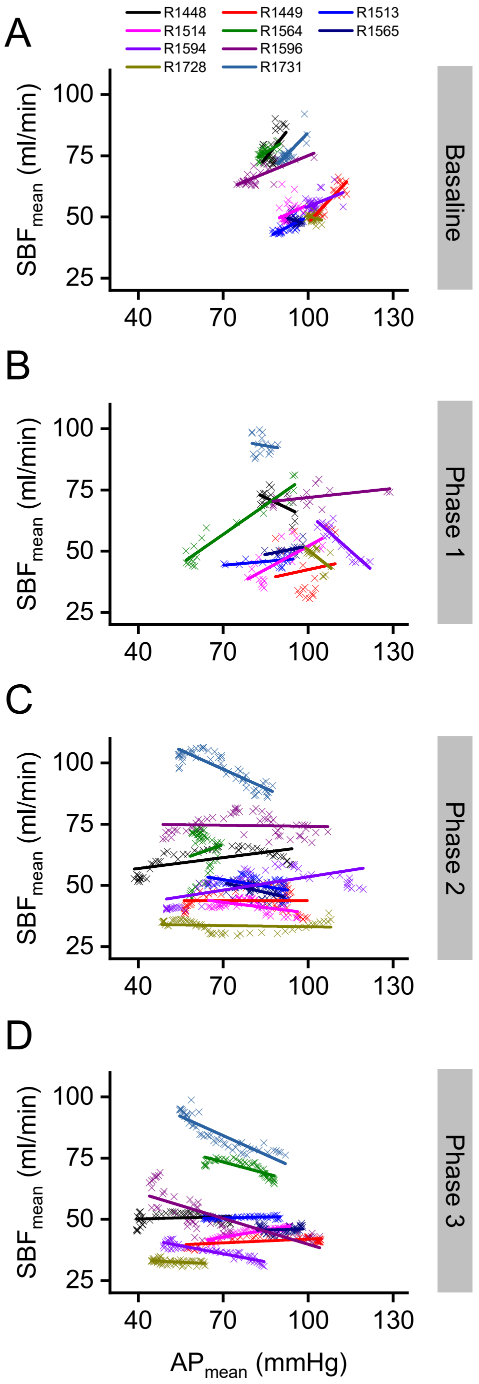

Figure S8. Linear correlations between min-to-min measures of SBF_{mean} and AP_{mean} in selected time windows before and after LPS. Baseline: 30 min that preceded the injection of LPS; phase 1: 10-30 min post-LPS; phase 2: 31-70 min post-LPS; phase 3: 71-120 min post-LPS. Data from different rats are presented in different colors.

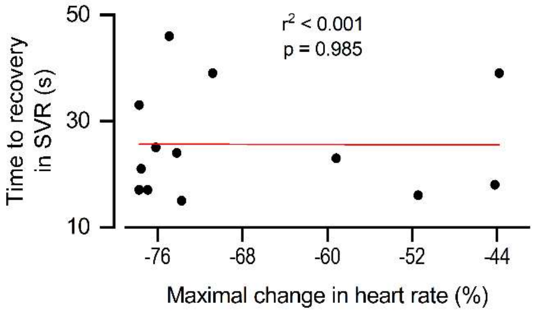

Figure S9. Lack of correlation between the time to recovery in SVR and the baroreflex-mediated heart rate response in PHE-injected rats. The data were subjected to linear regression.

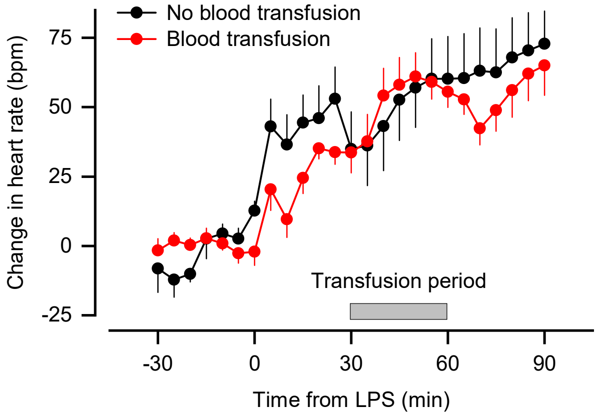

Figure S10. Autologous blood transfusion did not induce any sign of volume expansion, including reflexive bradycardia. The figure shows the time-course of the LPS-induced changes in heart rate in rats that received or did not receive the transfusion. The transfusion period is indicated. Data are expressed as means ± SEM. Groups consisted of 7-8 rats.
